# N-acetylcysteine (NAC) and Its Role in Clinical Practice Management of Cystic Fibrosis (CF): A Review

**DOI:** 10.3390/ph15020217

**Published:** 2022-02-11

**Authors:** Marta Guerini, Giorgia Condrò, Valeria Friuli, Lauretta Maggi, Paola Perugini

**Affiliations:** 1 Department of Drug Sciences, University of Pavia, Via Taramelli 12, 27100 Pavia, Italy; giorgia.condro01@universitadipavia.it (G.C.); valeria.friuli@unipv.it (V.F.); lauretta.maggi@unipv.it (L.M.); paola.perugini@unipv.it (P.P.); 2 Etichub, Academic Spin-Off, University of Pavia, Via Taramelli 12, 27100 Pavia, Italy

**Keywords:** cystic fibrosis, N-acetylcysteine, pseudomonas aeruginosa, biofilm, oxidative stress, lung diseases

## Abstract

N-acetylcysteine is the acetylated form of the amino acid L-cysteine and a precursor to glutathione (GSH). It has been known for a long time as a powerful antioxidant and as an antidote for paracetamol overdose. However, other activities related to this molecule have been discovered over the years, making it a promising drug for diseases such as cystic fibrosis (CF). Its antioxidant activity plays a key role in CF airway inflammation and redox imbalance. Furthermore, this molecule appears to play an important role in the prevention and eradication of biofilms resulting from CF airway infections, in particular that of *Pseudomonas aeruginosa*. The aim of this review is to provide an overview of CF and the role that NAC could play in preventing and eliminating biofilms, as a modulator of inflammation and as an antioxidant, restoring the redox balance within the airways in CF patients. To do this, NAC can act alone, but it can also be used as an adjuvant molecule to known drugs (antibiotics/anti-inflammatories) to increase their activity.

## 1. Introduction

Cystic fibrosis (CF) is an autosomal recessive disease with a systemic involvement, mainly affecting the respiratory and intestinal systems. This alteration results from a mutation in a gene discovered in 1989 on the long arm of chromosome 7 (7q31.2). This gene codes for a protein called the Cystic Fibrosis Conductance Regulator (CFTR), consisting of 1480 amino acids (170 KDa). The first symptoms of CF typically occur in childhood. CF is the main cause of chronic respiratory disease in children and is responsible for most pancreatic failures. This disease gives rise to multivarious manifestations, such as dehydration with salt loss, nasal polyposis, pansinusitis, rectal prolapse, pancreatitis, cholelithiasis, chronic liver disease, intestinal obstruction, male infertility, and diabetes [1,2]. Mutations in this gene are numerous; 2000 mutations have been counted to date, and their frequency varies by region. CF is most common in Caucasians in Northern Europe, North America, Australia, and New Zealand. The incidence varies between studies but is generally close to 1/3500 live births. CF is much less common in African American (1/17,000 live births) and Asian (1/90,000 live births) populations [3].

**Figure 1:**
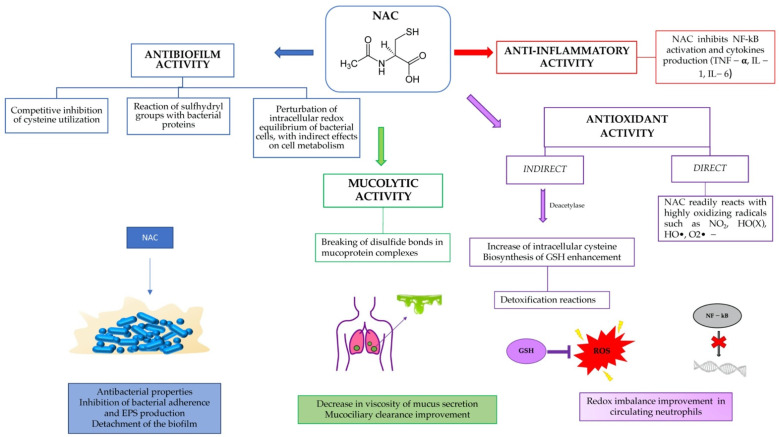
Summary of NAC activity in cystic fibrosis patients.

The aim of this review was to show the potential of a well-known and safe molecule, such as N-acetylcysteine, in preventing and eliminating biofilms, as a modulator of inflammation and as an antioxidant, restoring the redox balance within the airways in CF patients (Figure 1).

## 2. Literature Search Methodology

Literature searches were conducted using the PubMed database, entering keywords such as “N-acetylcysteine,” “cystic fibrosis,” “biofilm,” “respiratory tract infections,” and “*Pseudomonas aeruginosa*”. The WIPO platform has also been used, inserting as keywords “N-acetylcysteine,” and “transdermal administration.”

The authors examined the resulting lists of abstracts, excluding those that did not fit within the scope of the present work.

### 2.1. CFTR Protein

The CFTR protein is a single polypeptide chain (1480 amino acids), which plays a key role in the transepithelial transport of electrolytes and fluids. It functions both as a chlorine channel regulated by cyclic AMP and as a regulator of other ions. This protein consists of two transmembrane domains (each containing six α-helices), both of which are followed by a cytosolic nucleotide-binding domain. The former is followed by a regulatory R domain, whose phosphorylation by PKA is required to stimulate channel activity. The two transmembrane domains form a channel through which chlorine passes. Nucleotide binding to the channel leads to its opening, while the intrinsic ATPase activity of the channel itself leads to its closure [3]. The CFTR protein can therefore regulate multiple ion channels and cellular processes through its nucleotide binding domains. The epithelial sodium channel (ENaC) is normally inhibited by CFTR, but in CF, ENaC activity increases significantly by increasing sodium uptake through the apical membrane. The only exception is the human sweat ducts, where ENaC activity decreases due to CFTR mutations. Therefore, a hypertonic luminal fluid is formed that contains both a high sweat chloride and sodium content.

The functions of CFTR are tissue specific. In the sweat ducts it guides the reabsorption of salts, while in the intestinal epithelium, pancreatic ducts, and respiratory tract it promotes the secretion of ions and bicarbonate, and it is the most important pathway for the active secretion of luminal chloride. CFTR mutations cause reduced chloride secretion in the lumen and increased sodium uptake, which leads to passive reabsorption of water from the lumen and a depletion of the water content of the superficial fluid layer lining the mucosal cells [1]. Since 1989, many CFTR mutations have been identified, which can be grouped into six classes according to their effect:

Class I: in which mutations are associated with defective protein synthesis and there is a complete lack of CFTR protein on the apical surface of epithelial cells;

Class II: in which mutations are associated with an abnormal protein processing. The protein is not completely folded and glycosylated. The most common mutation (in about 70% of CF patients worldwide) is the deletion of three nucleotides coding for phenylalanine at amino acid position 508 (ΔF508);

Class III: in which mutations are associated with defective regulation of the protein, preventing ATP binding and hydrolysis;

Class IV: in which mutations are associated with protein production with decreased chlorine conductance;

Class V: in which mutations are associated with reduced protein production; and

Class VI: in which mutations are associated with altered regulation of separate ion channels [2,3].

### 2.2. Lung Pathophysiology

The lung is divided into two macro-regions, the conductive zone, whose function is to let air in and out, and the respiratory zone, which is used for gas exchange. The conductive zone includes the nasal cavity, the nasopharynx, the trachea, the bronchi, and the bronchioles, and is characterized by a ciliated respiratory epithelium covered by a dense mucus with a watery hypophase. From the trachea to the air sacs, there are 23 generations. As the subdivisions continue, the diameters decrease. Generation 16 is considered the last of the conductive zone; generation 17 is the first of the respiratory zone. The epithelium of the respiratory zone consists of a cell monolayer made up of four main cells: basal, ciliated, brush, and goblet cells. Finally, the alveolar epithelium is characterized by type I (96%) and type II pneumocytes [4].

The air surface liquid (ASL) consists of a 7–30 μm thick layer of fluid surrounding the base of the cilia, the periciliary fluid (PCL), and an overlying layer of mucus. The PCL is a polyanionic gel that provides a low-viscosity environment, preventing adhesion and the formation of mucus plugs. This layer has the function of trapping pathogens and inhaled particles through the mucin network, and removing them through ciliary clearance, which is the airways’ main innate defense mechanism against infection by inhaled bacteria.

CFTR’s role in chlorine secretion and sodium reabsorption underlies the regulation of ASL, and a defect in this protein leads to ASL volume depletion and mucus dehydration. This results in stagnant mucus that is no longer carried away by clearance. Mucus plugs create a tightly meshed mucin network that limits the rate of bacterial spread and the rate of neutrophil arrival, providing an ideal environment for bacterial biofilm formation. In addition, there is a reduction in intraluminal pH, which leads to mucin precipitation resulting in obstruction of the ducts and trapping of bacteria into the mucin [1]. Overlapping infections give rise to severe chronic bronchitis and bronchiectasis. The infection that characterizes CF airways involves the mucus layer rather than epithelial or airway wall invasion.

*Staphylococcus aureus*, *Hemophilus influenzae*, and *Pseudomonas aeruginosa* are the three most common organisms responsible for pulmonary infections. The mucoid phenotype of *P. aeruginosa*, characterized by the production of alginate, is particularly common. It has recently been shown that O_2_ tension is very low in CF mucus, and adaptation to hypoxia is an important feature of the physiology of CF bacteria. *Burkholderia cepacia* is also recovered in CF sputum. This bacterium is very resistant, and infection with this organism has been associated with fulminant illness, longer hospital stays, and increased mortality (”cepacian syndrome”). Other pathogens include *Stenotrophomonas maltophila*, *Alcaligenes xylosoxidans*, *B. gladioli*, *Escherichia coli*, *Klebsiella*, *Burkholderia multivorans*, *Achormobacter xyloxidans*, and the *Mycobacterium abscessus* complex [5].

### 2.3. Biofilm in CF Airways

Bacteria and fungi exist both as planktonic cells (in a more dangerous state) and as biofilms. Planktonic cells are either free single cells or organized in small chains (streptococci) or groups (staphylococci). They are not protected by toxic substances or bacteriophages. Biofilm, on the other hand, is characterized by aggregated cells protected by a self-produced matrix, which may also contain substances taken from the surrounding environment. These aggregates can adhere to both natural and artificial surfaces. The bacteria within the biofilm are 1000 times more resistant to both antibiotics and the host’s immune system. According to the National Nosocomial Infections Surveillance System, *Pseudomonas aeruginosa* is the third most common pathogen associated with hospital-acquired infections [6,7] and *Pseudomonas aeruginosa* infections in patients with cystic fibrosis were the first to be described in medicine. These infections originate in the paranasal sinuses and spread by inhalation with inspiration. 

**Figure 2:**
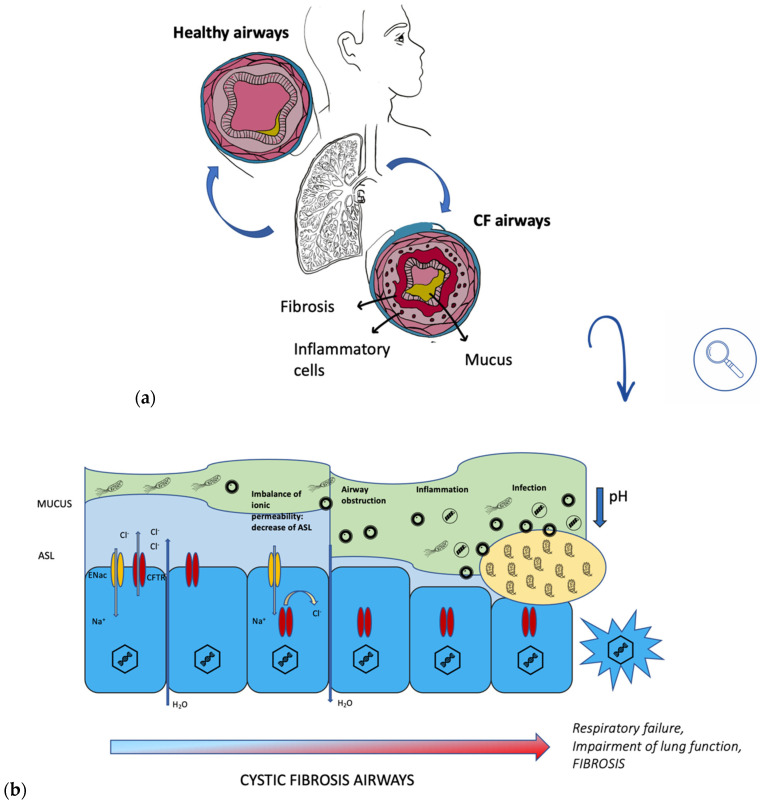
(**a**) comparison between healthy airways and CF airways; (**b**) progression of CF in airways.

During a lung infection, polymorphonuclear leukocytes (PMN) are recruited from the alveoli and brought into the conductive area. Here, excess PMN during phagocytosis of bacteria activates a metabolic process fueled by oxygen consumption, ROS formation, release of PMN-DNA, and PMN-proteases [8]. Sputum thus becomes slimy and anaerobic (Figure 2). The presence of PMN characterizes both the biofilm’s surroundings in the anaerobic zone, causing tissue damage, and the conductive zone, causing airway obstruction. The biofilm matrix of *P. aeruginosa* is characterized by the presence of alginate, extracellular DNA (eDNA), lipopolysaccharide (LPS), actin, and PMNs [9]. Overproduction of alginate, an exopolysaccharide composed of mannuronic and glucuronic acid monomers, is due to the mutation of a gene (*mucA*), resulting in the mucoid phenotype of *Pseudomonas aeruginosa* [5]. *MucA* encodes for an antisigma factor AlgT, which is required for the expression of the alginate biosynthetic operon. Alginate is a very important factor to consider, as it has a protective function in what is a dangerous environment for bacteria, which are constantly under attack from oxidative stress and from the host’s immune system. Therefore, the appearance of this strain gives rise to a much more serious infection than that resulting from the non-mucoid phenotype. It is important to note that *P. aeruginosa* biofilm is found within the mucus and not on the surface epithelium of non-respiratory bronchi and bronchioles [9].

## 3. N-acetylcysteine

N-acetylcysteine (NAC) (Figure 1) is the acetylated derivative of the amino acid L-cysteine and a precursor to the antioxidant glutathione. This molecule is one of the smallest drug molecules (19 atoms, MW 163.2) and has been used for decades to treat acetaminophen overdose and to dissolve mucus in the respiratory tract. However, its functional versatility and its ability to interact with different biochemical and molecular pathways have made it the subject of several in vitro and in vivo studies over the years, aimed at resolving various types of disorders and diseases. In view of this, NAC has been introduced into clinical practice for the treatment of pulmonary and cardiovascular diseases, psychiatric disorders, infectious diseases, rheumatoid arthritis, and plasma hyperlipoproteinemia [10,11,12]. Recently, a possible use of this molecule in SARS-CoV-2 infections has also been speculated [13].

This molecule is characterized by a pK_1_ (−SH) of 9.85 and a pK_2_ (COO−) of 3.31. There are three different forms of the molecule: NACH_2_, the neutral species (pH < 3.3); NACH^−^, the monoanionic species (3.3 > pH > 9.85); and NAC^2−^, the dianionic species (pH > 9.85).

### 3.1. Routes of Administration

#### 3.1.1. Oral Administration of NAC

The first pharmacokinetic parameters related to oral administration of NAC were attributed to the work of Borgström et al. [14] in which 10 healthy volunteers were considered. Four different formulations were administered: an effervescent tablet (Mucomyst), a granulate to be dissolved in water (Fabrol), a fast-acting tablet (NAC-Plain), and a slow-release tablet (NAC-SR). The authors showed that the mean residence time (MRT), i.e., the average transit time of the drug molecule through the body, of the three fast-release formulations was very similar, indicating that the absorption process had not been affected. The MRT of NAC-SR, on the other hand, was higher than that of the other oral formulations. The authors also showed that oral bioavailability varied between 6% and 10%, with the slow-release tablet having the lowest availability and the fast-dissolving tablet having the highest. However, it should be noted that the study conducted by Borgström et al. aimed to test the concentration of NAC in plasma samples present in a reduced form and mixed with other disulfide species, so it did not consider the protein-bound fraction of the drug.

Olsson et al. [15] assessed the pharmacokinetic by orally administering the N-acetylcysteine to six healthy volunteers, in the form of a 400 mg tablet. The results showed a bioavailability of reduced NAC of 4.0% (2.3–7.2%) and a bioavailability of total NAC (t½: 6.25 h) of 9.1% (4.8–13.1%). Furthermore, after about 60 min from the administration, it was noticed that the amount of NAC covalently bound to proteins in plasma increased, reaching 50% after 4 h. Subsequently, a reduction of the same data was observed to be approximately 20% after 12 h from the administration. The lower bioavailability of NAC after the oral administration and the difference between the reduced and total form are justified by the huge hepatic first-pass effect to which the substance is subject, and by its rapid oxidation, even before reaching systemic circulation. In addition, oral NAC undergoes gastrointestinal metabolism, where it is broken down (deacetylated) into cysteine. However, oral administration of NAC is characterized by rapid absorption. Indeed, the prevalence of NACH_2_ at pH < 3.3 allows rapid passive diffusion across the membrane from the gastric fluid, reaching peak plasma concentration in 90 min [16].

Decaro et al. [17] demonstrated that there are no significant pharmacokinetic differences when the drug is taken in a single dose of 600 mg or in multiple doses (i.e., 200 mg) administered multiple times per day. Oral administration does not consider dosage limits (up to 2 g per day), which generally vary according to the requirements of national authorities, because of the high tolerability of the drug. Side effects resulting from this route of administration mainly concern nausea, vomiting, diarrhea, rhinorrhea, and tachycardia [18,19]. The dosage of NAC is directly related to the treatment and the desired therapeutic effect. Oral intake of NAC represents a viable alternative route of administration that can solve the difficulties encountered in the inhalation of the substance, with respect to local irritative effects that may occur. In this case, the administration of high doses of NAC for a prolonged period has been shown to confer significant benefits through an indirect anti-inflammatory and antioxidant mechanism of action, essentially by NAC metabolites, such as cysteine [20]. However, Cotgreave et al. [21] demonstrated for the first time that after oral administration of 600 mg/day of NAC for 2 weeks, no N-acetylcysteine, free or bound, was found in bronchoalveolar lavage fluid (BALF) and the content of cysteine and glutathione in BALF was unaltered. At the same time, the content of Cys and GSH in plasma increased significantly.

#### 3.1.2. Intravenous Administration of NAC

The administration of NAC intravenously has been used mainly for the treatment of liver detoxification due to paracetamol poisoning, based on an FDA-approved three-bag regime. Initially, 150 mg/kg of NAC are administered in 200 mL of 5% dextrose for 60 min (first bag); then, 50 mg/kg are administered in 500 mL of 5% dextrose for 4 h (second bag); finally, 100 mg/kg are administered in 1000 mL of 5% dextrose for 16 h (third bag). Olsson et al. [15] investigated the effects of 200 mg/mL of NAC, diluted 1:10 in a saline solution (NaCl 0.9%) and infused over about 1 min. The kinetic curve, obtained by the correlation between plasma concentration of NAC and time from administration, achieved a high concentration of the drug (C_max_ approximately of 121 µM) that rapidly decreased in a triphasic manner, or biphasic in the case of reduced NAC. The volume of distribution was 0.47 l·kg^−1^ (0.59 l·kg^−1^ for reduced NAC), with a half-life of 5.58 h (1.95 h for reduced NAC), an MRT of 4.45 h; the total body clearance was 0.11 l·h^−1^·kg^−1^ (0.84 l·h^−1^·kg^−1^ for reduced NAC), and only 30% of the drug was excreted by renal clearance. The same authors also showed that after 60 min from administration, the concentration of NAC bound to proteins increased. Specifically, the percentage of drugs in the protein-bound form was 50% after 4 h, which decreased to 20% after 12 h.

The reported values of the volume distribution and the total body clearance confirmed the results previously obtained by Borgstrom et al. [14], who had analyzed samples of deproteinized plasma to investigate the effects of NAC administered intravenously over 5 min at the dose of 600 mg. The differences between the two studies concerned the half-life (5.58 h was detected by Olsson et al., while 2.27 h was detected by Borgstrom et al.) and the residence time (MRT) of the drug (4.45 h was detected by Olsson et al., while 1.62 h was detected by Borgstrom et al.) and they were probably due of the different analytical techniques used by the respective authors. A study conducted by Prescott et al. [22] showed the independence of the kinetics of NAC from the dose, reporting pharmacokinetics results that were approximately consistent with the previous studies. Moreover, they noted that there were no differences in NAC clearance between patients with liver damage and patients without.

A recent study [23] assessed enteral bioavailability, confirming previous findings [14,15].

Another important aspect of intravenous administration of NAC concerns the side effects that can occur during infusion of the drug, such as bronchospasm, hypotension, flushing, urticaria, and angioedema, which could be more serious or even fatal after an overdose of NAC (i.e., >3 g/day). However, compared to the oral administration of NAC, the intravenous NAC assures a higher plasma concentration of the drug, although it is not possible via either of these two routes to detect increased levels of the NAC or its metabolites in the BALF or lung tissue [24,25].

#### 3.1.3. Inhaled Administration of NAC

The inhaled administration of NAC is generally related to a mucolytic activity, in contrast to NAC oral administration, which is mainly linked to an antioxidant activity. In fact, this molecule, as well as other thiol derivatives, acts mainly on the lower respiratory tract to loosen the mucus, as the main target of the drug is the mucin. However, NAC is a very acidic compound (pH 2.2), and when inhaled it causes cough and bronchospasm. It has been suggested that induction of cough by inhaled NAC, rather than mucolytic activity, may explain any beneficial effect on expectoration (for example, in the treatment of cystic fibrosis, promoting sputum and reducing inflammation) [25]. Some previous studies showed that inhaled NAC is also effective on oxidative stress, finding that patients with higher oxidative stress may be good responders to inhaled NAC therapy, as the glutathione replenished by NAC inhalation can reverse an oxidant–antioxidant imbalance [26,27].

#### 3.1.4. Transdermal Administration of NAC

Transdermal delivery of NAC is rare. Only one study has outlined the cutaneous permeability of this molecule [28] and only a few studies have described the transdermal delivery of NAC. Bartek et al. evaluated transdermic absorption in rats, rabbits, pigs, and men of NAC labelled with C^14^ and S^35^, as a percentage of urine radioactivity after 5 days from index application. The partition coefficient of NAC was calculated to be 0.0004, between heptane and a pH 7.4 buffer. NAC was reported to be a weak penetrant in all species. The maximum percentage of absorption was reached in the pigs with a value of 6%, with lower values for other species (rats 3.50%, men 2.43%, rabbits 1.98%). These results were calculated assuming that both the excretion and distribution of NAC were equal after intravenous and transdermal administration. The authors reported 80% total radioactivity excreted in urine 24 h after intravenous administration of the index.

The main limitation of this study was the lack of consideration of factors that could influence the rate of penetration, such as the anatomical site of application, temperature, humidity, the nature of the vehicle, and the presence or absence of disease. In a patent in 2003, Hoek et al. described the invention of transdermal patches to administer NAC, encompassing salts, prodrugs, and metabolites as a mucolytic agent. The patches involved were the reservoir, matrix, drug-in-adhesive, multi-laminate type, and iontophoretic type.

To achieve the desirable therapeutic effect, the reservoir and the multi-laminate type were considered the best choices. However, one or more penetration enhancers were recommended to increase the amount of N-acetylcysteine. The penetration of NAC was investigated through pig skin using Franz diffusion cells, preparing four different solutions within N-acetyl-L-cysteine, with increases in the extent of NAC penetration shown in the following order: propylene glycol, water, ethanol, and propylene glycol containing 5% of azone. In this patent, the authors also underlined that NAC, being hydrophilic, has a limited extent of penetration, due to the low partition coefficient between lipids and water. Therefore, a modification of the carboxylic group and/or the thiol group could reduce hydrophilicity. Moreover, pro-drugs of N-acetylcysteine could also avoid or decrease skin metabolism.

An innovative way to deliver drugs transdermally is with the formulations of microneedles, which involve microscopic applicators to vehiculate high-molecular-weight molecules through the skin, bypassing the barrier of the stratum corneum.

Friden et al. [29], in his patent, described this modern delivery method. Peptides were administered, microporating the skin and adding iontophoresis. In other words, the microneedles system was combined with another modern technique to increase the penetration of peptides.

Iontophoresis involves the application of an electromotive force to drive ions through the stratum corneum and into the epidermal/dermal layers of the skin. The advantages of these systems are controlled release and the possibility of interrupting the administration simply by stopping the current and/or by removing the patch from the skin. In this work, N-acetylcysteine, despite being a tripeptide, was used as a penetration enhancer inside the formulation.

Another work, by Vazquez et al. [30], described the formulation of dissolving polymeric microneedles to deliver gentamicin transdermally for the treatment of possible serious bacterial infections (PSBI); however, N-acetylcysteine was used only as an excipient to increase the penetration of this ingredient.

### 3.2. Mechanism of Action

#### 3.2.1. Antioxidant Activity

The redox potential of the NAC thiol-disulfide couple (RSH-RSSR) is relatively high, making NAC a powerful agent capable of donating hydrogen radicals or acting as nucleophilic [19].

Several studies confirmed the in vitro and in vivo efficacy of NAC [16,31,32,33]. However, it is important to stress that there is a profound difference between them, i.e., in in vivo activity, there is a more complex situation due to the presence of other antioxidants, enzymatic or not, molecules, or substrates. The main oxidant species involved are H_2_O_2_, ONOOH, NO_2_, HO(X), HO•, and O_2_•−. The activity of NAC as an antioxidant, as well as all other antioxidant molecules, depends on the reaction rate, which should be higher than the reaction rate of the endogenous antioxidant molecules and much higher than that of the other substrates. The reaction rate is the product of the concentration of the molecule in which the oxidant is produced and the rate constant of the reaction of the antioxidant towards the oxidizing compound. Comparing the pKa of NAC (9.52), GSH (8.83), and Cys (8.30), NAC is the weakest antioxidant [34]. However, a high concentration of Cys cannot be used as a GSH precursor because it is toxic, and because of its rapid metabolism and oxidation [35].

NAC indirect antioxidant activity is due to the capability of the molecule to enter inside the cell and form glutathione (GSH). A key requirement for a substance to express its therapeutic effect is its ability to permeate biological membranes and be absorbed. To do this, most substances pass through passive transport, a diffusion process in which the driving force is the concentration gradient. Passive transport is influenced by several factors, including the pH, which is responsible for the ionized form of an ionizable compound. In this regard, at physiological pH (7.4), NAC is negatively charged, and the neutral form (NACH_2_) constitutes as little as 10^−3^% of total NAC concentration, while it has a neutral charge in acid pH conditions (pH < 3.3). This allows the passive diffusion of the substance from the gastric compartment to the bloodstream [19]. Here, NAC undergoes a process of deacetylation and consequent production of metabolites, such as cysteine, cystine, and sulfites, which will then support the biosynthesis of glutathione.

GSH is normally involved in numerous detoxification reactions, in which it functions as a substrate or cofactor in detoxifying cellular enzymes such as glutathione reductase, glutaredoxin, glutathione peroxidase, peroxidin, glyoxalase 1 and 2, glutathione transferase, and membrane-associated proteins in eicosanoid and glutathione metabolism (MAPEG). Decreased GSH is linked to aging and a wide range of diseases, especially in conditions of severe and prolonged oxidative stress and in neurodegenerative disorders [34].

#### 3.2.2. Disulfide Breaking Agent

In this mechanism, the reaction involved is a S_N_2, in which one bond is broken and one bond is formed synchronously, in a single reaction step. NAC thiolate binds the central sulfur of the disulfide and the leaving thiol is released via a trisulfide-like transition state structure.

NAC has a stronger disulfide breaking activity than other thiol compounds, such as cysteine and GSH, because of the relation between the rate of the thiol-disulfide interchange reaction and the nucleophilicity of the thiolate. The N-acetyl group and the carboxylated group of NAC stabilize the high electron density of the thiolate, increasing the nucleophilicity of the thiol. Hence, in contrast with antioxidant activity, which is related to SH acidity (in order, Cys > GSH > NAC), the reducing ability is related to SH basicity (in order, NAC > GSH > Cys).

Mucus is a substance characterized by 95% water and 5% glycoproteins, enzymes, salts, lipid, DNA, and cellular debris [36], produced by goblet and serous cells and secreted in reaction to an inflammatory response [37]. Mucin polymers, the main substances responsible for gel forming, are characterized by cysteine-rich regions that form internal cross-links upon oxidation. In healthy lungs, mucus has a low density and consequently it is easily transported. In inflammatory lung diseases, there is oxidation of the inner thiols of cysteine, which can provide the mucus with antioxidant effects, but can also generate disulfide cross-links between the inner domains of cysteine, leading to a strongly cross-linked mucus that is not easy to transport and can cause airway obstruction and lung infections [38].

#### 3.2.3. Antibiofilm Activity

Several previous works testified to the activity of NAC both as an antibacterial and as an anti-biofilm of different species, particularly against the biofilms of *Pseudomonas aeruginosa* (Table 1) [39,40,41,42,43,44,45,46,47,48,49,50,51].

The mechanism of action is not yet well known; however, the action of NAC against biofilms can be analyzed under different aspects: (i) antibacterial properties; (ii) detachment of the biofilm; and (iii) inhibition bacterial adherence and extracellular polysaccharides (EPS) production. With respect to the first point, NAC is considered to be a non-antibiotic drug, but one with antibacterial properties. NAC at pH higher than its pKa can penetrate the biofilm matrix and eventually kill 100% of the bacteria embedded in the biofilm [52,53]. The microcolonies of killed bacteria swell in size and are passively released. However, NAC used intravenously or in the presence of blood increases bacterial biofilm formation, rather than inhibiting it [54]. NAC and GSH have been shown to dramatically increase the killing of *Staphylococcus aureus* neutrophils in vitro. One possible explanation is that they reduce the concentration of extracellular NO.

The antimicrobial and antibiofilm activity of NAC was also tested against a collection of strains of *Stenotrophomonas maltophilia* (*n* = 19) and the *Burkholderia cepacia* complex (Bcc) (*n* = 19), which are recognized as increasingly frequent strains in CF patients. Minimum inhibitory concentrations (MIC) and minimum bactericidal concentrations (MBC) ranged from 16 to 32 mg/mL and 32 to >32 mg/mL, respectively [51]. *Pseudomonas aeruginosa* proved to be the most susceptible bacterium to the action of NAC [40]. The mechanism of action of NAC as a bactericide could lie in the competitive inhibition of cysteine use, or in the reaction with bacterial cell proteins through its sulfhydryl groups. However, the structure and physiology of the biofilm allow it to be much more resistant to the action of antimicrobics than planktonic cells. During the different studies on how NAC could act, its ability to inhibit biofilm adhesion to the substrate and reduce EPS production was highlighted [40,41,42].

Olofsson et al. [43] studied 10 different bacterial strains and concluded that NAC inhibits the production of EPS at 0.25 mg/mL. EPS contain water and minerals and provide nourishment for bacteria, therefore a degradation of EPS leads to a lower defense of the bacterium. The activity of NAC is mainly provided by the -SH group, which plays a role in both free radical scavenging and the intra- and intermolecular destruction of disulfide groups of proteins. Therefore, the destruction of EPS by NAC can occur in different ways: the sulfhydryl group can destroy the disulfide bonds of the bacterial enzymes involved in the production of EPS, and the antioxidant action can indirectly interfere with cell metabolism and EPS production [43].

## 4. The Role of NAC in Cystic Fibrosis

Oxidative stress is now recognized as one of the predisposing factors in the development of diseases such as CF. An adequate level of GSH is essential to combat excessive ROS production. Inflammation and infection are hallmarks of CF lungs and are closely linked to CFTR dysfunction. In addition, many pathogenic bacteria have evolved not only to survive, but also to thrive in an environment characterized by the oxidative stress caused by infection [55].

Lipopolysaccharide (LPS) is an important pro-inflammatory glycolipid component that characterizes the outer membrane of gram-negatives, such as *Pseudomonas aeruginosa*. The presence of LPS induces an increase of neutrophils, macrophages, and cytokines in sputum and bronchoalveolar lavage fluid (BALF) and induces an increase of ROS [56]. NAC can act in this type of environment both as an antioxidant and as an anti-inflammatory in a concentration-dependent manner [54]. In fact, the presence of LPS leads to the release of neurokine A (NKA) [56], which plays an important role in triggering inflammation. In this regard, Calzetta et al. demonstrated that NAC has an anti-inflammatory effect at both high and low concentrations, and that it has the power to act against LPS by modulating both NKA and IL-6 levels [57].

Moreover, CF airway inflammation is due to an excessive neutrophil recruitment, triggered by extrinsic and intrinsic factors, and by an imbalance between reduced concentrations of anti-inflammatory molecules (IL-10, lipoxins, chemotaxis inhibitors, and neutrophil activators) and higher levels of pro-inflammatory proteins (calprotectin). Malabsorption of dietary antioxidants in the gut and the inability of cells with the CFTR mutation to efflux glutathione (GSH) play an important role in the systemic redox imbalance already exacerbated by the excessive release of oxidants by neutrophils. This inflammation is a self-amplifying process, with neutrophil-derived effectors promoting the exit of neutrophils from the bone marrow into the circulation and subsequently into the CF airways.

Thus, it can be established that neutrophils are the cellular link between redox imbalance and inflammatory imbalance [58].

N-acetylcysteine, as a GSH prodrug, could improve this imbalance by increasing GSH in blood neutrophils and decreasing neutrophils and elastase activity in CF airways. Since NAC has demonstrated long-term safety at high doses, its use in combination with other drugs (antibiotics/anti-inflammatories) could be a plausible solution to fight and prevent inflammation [59].

Mutated CFTR may be associated with an alteration of certain signal transduction pathways at the cellular level, such as that of NFkB (nuclear factor kappa-light-chain- enhancer of activated B cells). In the lung, NFkB is required for the transcription of several pro-inflammatory molecules, and it is overexpressed in CF. Reactive oxygen species (ROS) are activators of NFkB and the same stimulation of bacteria on the cell surface induces its activation [37]. The CFTR mutation is also associated with reduced production of PPAR (peroxisome proliferator-activated receptor), a transcription factor, which has an opposite action to NFkB and therefore a contrasting activity [58]. An influence of NAC on NFkB activation was observed, up to a concentration of 45 mM [37], which is a high concentration. However, NAC appears to have a cell type-specific action: in human bronchial epithelial cells, NAC inhibited silica-induced NFkB at a concentration of 5 mM [60].

These results show great promise because they demonstrate NAC activity at low concentrations.

Other research has suggested other mechanisms of action that may involve the effects of NAC on CFTR. Luciani et al. demonstrated an autophagic pathway for intracellular trafficking, dependent on CFTR function, which is restored by NAC treatment allowing normal CFTR maturation and trafficking to the cell surface [61]. Furthermore, Chen et al. found that NAC treatment ameliorated the overproduction of oxidants and subsequent cytokine overexpression, as they observed a decrease in Nrf-2 (nuclear regulatory factor)-dependent antioxidant responses in CF epithelia, resulting in an increase in hydrogen peroxide (H_2_O_2_), thus contributing to the overproduction of the inflammatory pro-cytokines IL- 6 and IL-8 [62]. In addition, hypochlorous acid and its derivatives (HOX: hypobromous acid, HOBr, hypo-thiocyanous acid, HOSCN) can play an important role in the pathophysiology of CF [63]. HOX is produced by activated neutrophils and monocytes through the activation of myeloperoxidase (MPO), which catalyzes the reaction between hydrogen peroxide and halides. These oxidants are bactericidal and disinfectant, aiding the human response against pathogens, and can also react with important biological molecules, inducing cytotoxic effects. High levels of MPO protein, increased halogenated proteins, and disulfide bonds are reported in the airway mucus of CF patients, suggesting that oxidation occurring from airway inflammation contributes to the formation of viscous, pathological mucus in the affected lungs. Thus, high concentrations of NAC in this condition, which sees a depletion of the -SH pool, may neutralize HOX species [34,64].

With respect to NAC anti-biofilm activity, Table 1 shows some examples of in vitro studies to evaluate the anti-biofilm power of NAC alone and in combination with antibiotics or anti-inflammatories.

To date, gram-negative bacilli (GNB) play a major role in CF, and over the years these GNBs have shown increasing resistance to antibiotics, limiting the treatment of this disease.

To cope with this resistance, physicians in clinical practice use different associations of aminoglycoside (tobramycin), polymyxin (colistin), and fluoroquinolone (ciprofloxacin) [65,66] to treat *P. aeruginosa*, or other less common gram-negative pathogens. Antibiotic efficacy, however, can be improved when used in combination with a non-antibiotic compound, such as NAC.

Synergy between compounds is defined as “when the minimal inhibitory concentration (MIC) of the individual compound is decreased significantly after the compounds are combined” [66].

From the literature, it is possible to notice the interaction between ciprofloxacin and NAC and their synergistic action (50%) in the biofilm detachment, as demonstrated by Zaho et al. [40], meaning that NAC and ciprofloxacin could be used together to treat *P. aeruginosa* biofilm. In this work, it is also clear that *P. aeruginosa* is more susceptible to NAC than are other strains and that there is another interesting synergistic effect with carbenicillin and tobramycin. In fact, carbenicillin MIC decreases from 16 µg/mL to 1 µg/mL in the presence of NAC.

Lea et al. [45] demonstrated that the resistance of the bacterium to ciprofloxacin fails with a combination of NAC and antibiotics. In a recent work, a significant decrease in planktonic and attached bacterial growth was observed using combinations of ciprofloxacin/colistin and NACH_2_. Significant reductions in CFU/mL in mature biofilms were also observed [67]. In addition, NAC has also been shown to antagonize colistin resistance mechanisms, especially against strains such as *S. malthopila* [68].

The effect of NAC with other anti-inflammatories was then studied, evidencing that the combination of NAC/diclofenac, NAC/ibuprofen, and NAC/ketoprofen increases biofilm detachment activity compared to single active ingredients alone [44]. These works, despite being in vitro, give great hope for using this molecule as an antibiotic or anti-inflammatory enhancer, through a local administration route such as the inhalation route, to have the greatest concentration of the active in the site of action. However, it would be appropriate to conduct in vivo studies to confirm this hypothesis, as there are only a few so far (Table 2).

## 5. Conclusions

With this work, we wanted to show the various roles that NAC can play in cystic fibrosis, not only used individually, but also as an aid to therapies that today are not very effective. Based on pharmacokinetic data, the best solution would be to administer NAC by inhalation, in order to bypass the first-pass effect and arrive directly at the site of action. In this way, the concentrations of the active would be decreased. Several works have also shown that intravenous administration of NAC does not change the GSH levels on BALF or in lung tissue.

Moreover, experimental data have shown that the concentrations needed to modulate an oxidant/antioxidant imbalance are much higher than those needed to elicit anti-inflammatory effects. Indeed, a low concentration of 200 mg/day orally is capable of reducing IL-6 levels, which are inversely correlated with NKA release [73]. This suggests that low concentrations of NAC can overcome neurogenic inflammation, a deleterious condition that supports the vicious circle between oxidative stress and inflammation in the bronchi.

To conclude, it is possible to establish that this molecule opens great hope in the treatment of this disease. However, there is still a lack of many in vivo studies on which to base this hypothesis.

## Figures and Tables

**Table 1:** Examples of in vitro NAC activity against biofilm.

Bacteria Strains	Effective NAC Concentration	Comments	Reference
*P. aeruginosa* ATCC 10145	0.5, 2 and 4 mg/mL	Biofilm prevention activity: reduction of biofilm growth by 19.42%, 20.87%, and 89.52% at NAC concentration of 0.5, 2, and 4 mg/mL.	[41]
*P. aeruginosa* PAO1	0.5–1 mg/mL	Combination of NAC and Ciprofloxacin shows synergy (50%) or no interaction (50%). Extracellular polysaccharides (EPS) production decreased by 27.64% and 44.59% at NAC concentrations of 0.5 mg/mL and 1 mg/mL.	[40]
*P. aeruginosa* ATCC 10145	2.5 mg/mL	NAC shows a higher inhibitory effect in bacterial adherence, combined with NSAIDs *.	[44]
15 strains of *P. aeruginosa* isolated from clinical specimens of patients with CSOM *; *P. aeruginosa* ATCC 35032 used as a quality control organism	2.5, 5 and 12.5 mg/mL	*P. aeruginosa* strains in the sessile (33–40%) and planktonic (13%) state demonstrates resistance to Ciprodex^®^ and ciprofloxacin. When NAC ≥0.5% was used in isolation or as an adjunct to either of these medications, no resistance was found in the sessile or planktonic state among all 15 strains.	[45]
2 strains of *P. aeruginosa* isolated from urine samples and stent segment	2–4 mg/mL	NAC at 2 mg/mL reduces more than 60% of biofilm synthesis, and at 4 mg/mL reduces 76.7% of biofilm synthesis. Ciprofloxacin/NAC combination reveals a reduction power of 94–100%	[46]
*P. aeruginosa* ATCC 17334	0.5–2 mg/mL	0.5 mg/mL of NAC inside microstructured lipid carrier produce a reduction of 64.74% ± 6.2% biofilm growth, while the same amount of placebo MLC (without NAC) produced a reduction of only 8.57% ± 1.2% (*p* < 0.05). NAC concentration of 2 mg/mL is able to reduce 83.74% ± 9.95% of biofilm, compared to a reduction of 10.53% ± 0.6% made by the same amount of placebo MLC (*p* < 0.05).	[47]
*P. aeruginosa* ATCC 15692	6 mg	NAC can enhance ciprofloxacin effect when used in combination with it in bone cement.	[48]
15 strains of *S. epidermidis*	0.003–8 mg/mL	A dose-related decrease in biofilm is observed: a decrease of 63%, 55%, 46%, 34%, 26%, and 26% is noticed in the presence of 0.25, 0.5, 1, 2, 4, and 8 mg/mL of NAC, respectively. The inhibitory effect of 2 mg/mL of NAC on slime formation was also verified by electron microscopy.	[49]
10 strains isolated from paper mill: *Acinetobacter baumannii*, *Acinetobacter lwoffii*, *Bacillus* sp., *Bacillus cereus*, *bacillus megaterium, bacillus subtilis, Enterobacter* *cloacae*, *Klebsiella pneumoniae*, *Pseudomonas mendocina*, *Staphylococcus warneri.*	0.25, 0.5, 1 mg/mL	Gram-positive strains appear more sensitive to NAC, which decreases bacterial adhesion and detaches bacteria from the stainless-steel surface. Moreover, NAC decreases EPS production in most bacteria tested.	[43]
Four strains of uropathogenic *Escherichia coli*	0.007–8 mg/mL	NAC (2 mg/mL) used in combination with fosfomycin (2000 mg/L) enhances the anti-biofilm effect with a reduction of 99.99% of viable cells.	[42]
*Enterococcus faecalis* ATCC *29212*	1.56–12.5 mg/mL	NAC results in bactericidal at pH 11 (MIC = 1.56 mg/mL; MBC = 12.5 mg/mL) and results able to eradicate *E. faecalis* biofilm.	[50]
*Stenotrophomonas maltophilia* and *Burkholderia cepacia* complex (Bcc)	8, 16, 32 mg/mL	Dose- and time-dependent antibiofilm activity of NAC was observed against the majority of *S. maltophilia* and *Bcc* strains tested. MIC S. maltophilia: 16–32 mg/mL; MIC Bcc: >32 mg/mL	[51]

* NSAIDs: non-steroidal anti-inflammatory drugs; CSOM: chronic suppurative otitis media.

**Table 2:** N-acetylcysteine studies in vivo on CF patients in literature.

Administration Route	Posology	NAC Activity	Comments	Reference
Oral	0.6 to 1.0 g three times daily, for 4 weeks	Inflammation modulator/antioxidant	Safe treatment; decrease of sputum elastase activity (*p* = 0.006); decrease of neutrophil burden in CF airways (*p* = 0.003); pulmonary function measures not improved.	[59]
Oral	900 mg three times/day for 24 weeks	Inflammation modulator/antioxidant	Lung function (FEV_1_ and FEF 25–75% remained stable or increased slightly in the NAC group but decreased in the placebo group (*p* = 0.02 and 0.02). Log_10_ HNE activity remained equal between cohorts (difference 0.21, 95% CI −0.07 to 0.48, *p* = 0.14).	[69]
Oral	200 mg three times/daily or 400 mg three times daily	Inflammation modulator/antioxidant	Patients with PEFR below 70% of predicted normal values showed a satisfactory significant increase in PEFR, FVC and in one second FEV during NAC treatment. No effect of NAC on ciliary activity was observed.	[70]
Oral	700 mg /daily (low dose) or 2800 mg/daily (high dose)	Inflammation modulator/antioxidant	High-dose NAC was a well-tolerated and safe medication. High-dose NAC did not alter clinical or inflammatory parameters. However, extracellular glutathione in induced sputum tended to increase on high-dose NAC.	[71]
Oral	2400 mg/ daily for 4 weeks	Inflammation modulator/antioxidant	A better lung function was observed in the NAC treated group with a mean (SD) change compared to baseline of FEV1% predicted of 2.11 (4.6), while a decrease was observed in the control group (change—1.4 (4.6)), though not statistically significant.	[72]

FVC: forced vital capacity; FEV: forced expiratory volume; HNE: human neutrophil elastase; PEFR: peak expiratory flow rate.

## Data Availability

Data is contained within the article.

## References

[1] Castellani C., Assael B.M. (2017). Cystic fibrosis: A clinical view. Cell. Mol. Sci..

[2] Boeck K.D. (2020). Cystic fibrosis in the year 2020: A disease with a new face. Acta Pediatrica..

[3] Bell S.C., Mall M.A., Gutierrez E., Macek M., Madge S., Davies J.C., Burgel P.R., Tullis E., Castaños C., Castellani C. (2020). The future of cystic fibrosis care: A global perspective. Lancet Respir. Med..

[4] Newman S.P. (2017). Drug delivery to the lungs: Challenges and opportunities. Ther. Deliv..

[5] O’Brien S., Fothergill J.L. (2017). The role of multispecies social interactions in shaping *Pseudomonas aeruginosa* pathogenicity in the cystic fibrosis lung. FEMS Microbiol. Lett..

[6] Pachori P., Gothalwal R., Gandhi P. (2019). Emergence of antibiotic resistance Pseudomonas aeruginosain intensive care unit: A critical review. Genes Dis..

[7] Waters V.J., Kidd T.J., Canton R., Ekkelenkamp M.B., Johansen H.K., LiPuma J.J., Bell S.C., Elborn J.S., Flume P.A., Van Devanter D.R. (2019). Reconciling Antimicrobial susceptibility testing and clinical response in antimicrobial treatment of chronic cystic fibrosis lung infections. Clin. Infect. Dis..

[8] Kolpen M., Hansen C.R., Bjarnsholt T., Moser C., Christensen L.D., van Gennip M., Ciofu O., Mandsberg L., Kharazmi A., Döring G. (2010). Polymorphonuclear leukocytes consume oxygen in sputum from chronic Pseudomonas aeruginosa pneumonia in cystic fibrosis. Thorax.

[9] Rossi E., La Rosa R., Bartell J.A., Marvig L.R., Haagensen J.A.J., Sommer L.M., Molin S., Johansen H.K. (2020). Pseudomonas aeruginosa adaptation and evolution in patients with cystic fibrosis. Nat. Rev. Microbiol..

[10] Dludla P.V., Tiano L., Louw J., Mxinwa V., Tiano L., Marcheggiani F., Cirilli I., Louw J., Nkambule B.B. (2019). The beneficial effects of N-acetyl cysteine (NAC) against obesity associated com- plications: A systematic review of pre-clinical studies. Pharmacol. Res..

[11] Ooi S.L., Green R., Pak S.C. (2018). N-Acetylcysteine for the Treatment of Psychiatric Disorders: A Review of Current Evidence. BioMed Res. Int..

[12] Gerry K. (2021). Schwalfenberg, N-Acetylcysteine: A Review of Clinical Usefulness (an Old Drug with New Tricks). J. Nutr. Metab..

[13] Shi Z., Puyo C.A. (2020). N-acetylcysteine to combat COVID-19: An evidence review. Ther. Clin. Risk Manag..

[14] Borgstrom L., Kgtgedal B., Paulsen O. (1986). Pharmacokinetics of N-Acetylcysteine in Man. Eur. J. Clin. Pharmacol..

[15] Olsson B., Johansson M., Gabrielsson J., Bolme P. (1988). Pharmacokinetics and Bioavailability of Reduced and Oxidized N-Acetylcysteine. Eur. J. Clin. Pharmacol..

[16] Rind L., Ahmad M., Khan M.I., Badrudeen, Akhtar J., Ahmad U., Yadav C., Owais M. (2021). An insight of safety, efficacy, and molecular docking study reportsof N-acetylcysteine and its compound formulations. J. Basic Clin. Physiol. Pharmacol..

[17] DeCaro L., Ghizzzi A., Costa R., Longo A., Ventresca G.P. (1989). Pharmacokinetics and bioavailability of oral acetylcysteine in healthy volunteers. Arzneim. -Forsch..

[18] Ershad M., Naji A., Vearrier D.N. (2019). Acetylcysteine. Eur. PMC.

[19] Salamon S., Kramar B., Marolt T.P., Poljska B., Milisav I. (2019). Medical and Dietary uses of N-acetylcysteine. Antioxidants.

[20] Harada D., Naito S., Hiraoka I., Otagiri M. (2002). In vivo kinetic analysis of covalent binding between N-acetyl-L-cysteine and plasma protein through the formation of mixed disulfide in rats. Pharm. Res..

[21] Cotgreave I.A., Moldeus P. (1987). Methodologies for the analysis of reduced and oxidized N-acetylcysteine in biological systems. Biopharm. Drug Dispos..

[22] Prescott L.F., Donovan J.W., Jarvie D.R., Proudfoot A.T. (1989). The disposition and kinetics of intravenous N-acetylcysteine in patients with paracetamol overdosage. Eur. J. Clin. Pharmacol..

[23] Teder K., Maddison L., Soeorg H., Meos A., Karjagin J. (2021). The Pharmacokinetic Profile and Bioavailability of Enteral N-Acetylcysteine in Intensive Care Unit. Medicine.

[24] Bridgeman M.M., Marsden M., Selby C., Morrison D., MacNee W. (1994). Effect of N-acetyl cysteine on the concentrations of thiols in plasma, bronchoalveolar lavage fluid, and lung tissue. Thorax.

[25] Tam J., Nash E.F., Ratjen F., Tullis E., Stephenson A. (2013). Nebulized and oral thiol derivatives for pulmonary disease in cystic fibrosis. Cochrane Database Syst. Rev..

[26] Muramatsu Y., Sugino K., Ishida F., Tatebe J., Morita T., Homma S. (2016). Effect of inhaled N-acetylcysteine monotherapy on lung function and redox balance in idiopathic pulmonary fibrosis. Respir. Investig..

[27] Calverley P., Rogliani P., Papi A. (2021). Safety of N-acetylcysteine at high doses in chronic respiratory diseases: A review. Drug Saf..

[28] Bartek J., Labudde J.A., Maibach H.I. (1972). Skin permeability in vivo: Comparison in rat, rabbit, pig and man. J. Investig. Dermatol..

[29] Friden P.M. (2009). Method of Enhancing Iontophoretic Delivery of a Peptide. U.S. Patent.

[30] González-Vázquez P., Larrañeta E., McCrudden M.T.C., Jarrahian C., Rein-Weston A., Quintanar-Solares M., Zehrung D., McCarthy H., Courtenay A.J., Donnelly R.F. (2017). Transdermal delivery of gentamicin using dissolving microneedle arrays for potential treatment of neonatal sepsis. J. Control. Release.

[31] Cazzola M., Calzetta L., Facciolo F., Rogliani P., Matera M.G. (2017). Pharmacological investigation on the anti-oxidant and anti-inflammatory activity of N-acetylcysteine in an ex vivo model of COPD exacerbation. Respir. Res..

[32] Ezeriņa D., Takano Y., Hanaoka K., Urano Y., Dick T.P. (2018). N-acetyl cysteine functions as a fast-acting antioxidant by triggering intracellular H2S and sulfane sulfur production. Cell Chem. Biol..

[33] Zhang H., Su W., Ying Z., Chen Y., Zhou L., Li Y., Zhang J., Zhang L., Wang T. (2018). N-acetylcysteine attenuates intrauterine growth retardation-induced hepatic damage in suckling piglets by improving glutathione synthesis and cellular homeostasis. Eur. J. Nutr..

[34] Aldini G., Altomare A., Baron G., Vistoli G., Carini M., Borsani L., Sergio F. (2018). N-Acetylcysteine as an antioxidant and disulphide breaking agent: The reasons why. Free Radic. Res..

[35] Rushworth G.F., Megson I.L. (2014). Existing and potential therapeutic uses for N-acetylcysteine: The need for conversion to intracellular glutathione for antioxidant benefits. Pharmacol. Ther..

[36] Calzetta L., Matera M.G., Rogliani P., Cazzola M. (2018). Multifaceted activity of N-acetyl-L-cysteine in chronic obstructive pulmonary disease. Expert Rev. Respir. Med..

[37] Cazzola M., Calzetta L., Page C., Rogliani P., Matera M.G. (2019). Thiol-Based drugs in pulmonary medicine: Much more than mucoltycs. Trend Pharm. Sci..

[38] Yuan S., Hollinger M., Lachowicz-Scroggins M.E., Kerr S.C., Dunican E.M., Daniel B.M., Ghosh S., Erzurum S.C., Willard B., Hazen S.L. (2015). Oxidation increases mucin polymer cross-links to stiffen airway mucus gels. Sci. Transl. Med..

[39] Kundukad B., Schussman M., Yang K., Seviour T., Yang L., Rice S.A., Kjelleberg S., Doyle P.S. (2017). Mechanistic action of weak acid drugs on biofilms. Sci. Rep..

[40] Zhao T., Liu Y. (2010). N-acetylcysteine inhibits biofilms produced by *Pseudomonas Aeruginosa*. BMC Microbiol..

[41] Guerini M., Perugini P., Grisoli P. (2020). Evaluation of the Effectiveness of N-Acetylcysteine (NAC) and N-acetylcysteine-cyclodextrins Multi-Composite in Pseudomonas aeruginosa Biofilm Formation. Appl. Sci..

[42] Marchese A., Bozzolasco M., Gualco L., Debbia E.A., Schito G.C., Schito A.M. (2003). Effect of fosfomycin alone and in combination with N-acetylcysteine on E. coli biofilms. Intern. J. Antimicrob. Agent.

[43] Olofsson A.C., Hermansson M., Elwing H. (2003). N-acetyl-L-cysteine affects growth, extracellular polysaccharide production, and bacterial biofilm formation on solid surfaces. Appl Env. Microbiol..

[44] Mohsen A., Gomaa A., Mohamed F., Ragab R., Eid M., Ahmed A.H., Khalaf A., Kamal M., Mokhtar S., Mohamed H. (2015). Antibacterial, anti-biofilm activity of some non-steroidal anti-inflammatory drugs and N-acetyl cysteine against some biofilm producing uropathogens. Am. J. Epidemiol. Infect. Dis..

[45] Lea J., Conlin A.E., Sekirov I., Restelli V., Ayakar K.G., Turnbull L., Doyle P., Noble M., Rennie R., Schreiber W.E. (2014). In vitro efficacy of N-acetylcysteine on bacteria associated with chronic suppurative otitis media. J. Otolaryngol. Head Neck Surg..

[46] El-Feky M.A., El-Rehewy M.S., Hassan M.A., Abolella H.A., Abd El-Baky R.M., Gad G.F. (2009). Effect of ciprofloxacin and N-acetylcysteine on bacterial adherence and biofilm formation on ureteral stent surfaces. Pol. J. Microbiol..

[47] Guerini M., Grisoli P., Pane C., Perugini P. (2021). Microstructured Lipid Carriers (MLC) Based on N-Acetylcysteine and Chitosan Preventing *Pseudomonas aeruginosa* Biofilm. Int. J. Mol. Sci..

[48] Onger M.E., Gocer H., Emir D., Kaplan S. (2016). N-acetylcysteine eradicates Pseudomonas aeruginosa biofilms in bone cement. J. Scann Microsc..

[49] Perez A., van Heeckeren A.M., Nichols D., Gupta S., Eastman J.F., Davis P.B. (2008). Peroxisome proliferator- activated receptor-gamma in cystic fibrosis lung epithelium. Am. J. Physiol. Lung Cell Mol. Physiol..

[50] Quah S.Y., Wu S., Lui J.N., Sum C.P., Tan K.S. (2012). N-Acetylcysteine Inhibits Growth and Eradicates Biofilm of Enterococcus faecalis. J. Endod..

[51] Pollini S., Di Pilato V., Landini G., Di Maggio T., Cannatelli A., Sottotetti S., Cariani L., Aliberti S., Blasi F., Sergio F. (2018). In vitro activity of N-acetylcysteine against *Stenotrophomonas maltophilia* and *Burkholderia cepacia* complex grown in planktonic phase and biofilm. PLoS ONE.

[52] Pintucci J.P., Corno S., Garotta M. (2010). Biofilm and infections of the upper respiratory tract. Eur. Rev. Med. Pharmacol. Sci..

[53] Volgers C., Benedikter B.J., Grauls G.E., Hellebrand P.H.M., Savelkoul P.H.M., Stassen F.R. (2017). Effects of N-acetyl-L-cysteine on the membrane vesicle release and growth of respiratory pathogens. FEMS Microbiol..

[54] Yin S., Jiang B., Huang G., Zhang Y., You B., Chen Y., Chen J., Yuan Z., Zhao Y., Li M. (2018). The interaction of N-acetylcysteine and serum transferrin promotes bacterial biofilm formation. Cell Physiol. Biochem..

[55] Reniere M.L. (2018). Reduce, induce, thrive: Bacterial redox sensing during pathogenesis. J. Bacteriol..

[56] Calzetta L., Luongo L., Cazzola M., Page C., Rogliani P., Facciolo F., Maione S., Capuano A., Rinaldi B., Matera M.G. (2015). Contribution of sensory nerves to LPS-induced hyperresponsiveness of human iso- lated bronchi. Life Sci..

[57] Calzetta L., Rogliani P., Facciolo F., Rinaldi B., Cazzola M., Matera M.G. (2018). N-Acetylcysteine protects human bronchi by modulating the release of T neurokinin A in an ex vivo model of COPD exacerbation. Biomed. Pharmacother..

[58] Jennings M.T., Flume P.A. (2018). Cystic fibrosis: Traslating molecular mechanisms into effective therapies. Ann. Am. Thorac. Soc..

[59] Tirouvanziam R., Conrad C.K., Bottiglieri T., Herzenberg L.A. (2006). High-dose oral N-acetylcysteine, a glutathione prodrug, modulates inflammation in cystic fibrosis. PNSA.

[60] Desaki M., Takizawa H., Kasama T., Kobayashi K., Morita Y., Yamamoto K. (2000). Nuclear factor-kb activation in silica-induced interleukin 8 production by human bronchial epithelial cells. Cytokine.

[61] Luciani A., Villella V.R., Esposito S., Brunetti-Pierri N., Medina D., Settembre C., Gavina M., Pulze L., Giardino I., Pettoello-Mantovani M. (2010). Defective CFTR induces aggresome formation and lung inflammation in cystic fibrosis through ROS-mediated autophagy inhibition. Nat. Cell Biol..

[62] Chen J., Kinter M., Shank S., Cotton C., Kelley T.J., Ziady A.G. (2008). Dysfunction of Nrf-2 in CF epithelia leads to excess intracellular H2O2 and inflammatory cytokine production. PLoS ONE.

[63] O’Donnell C., Newbold P., White P., Thong B., Stone H., Stockley R. (2010). A3-Chlorotyrosine in sputum of COPD patients: Relationship with airway inflammation. COPD.

[64] Hawkins C.L., Davies M.J. (2021). Role of myeloperoxidase and oxidant formation in the extracellular enviroment in inflammation-induced tissue damage. Free. Radic. Biol. Med..

[65] Thai T., Salisbury B.H., Zito P.M. (2021). Ciprofloxacin.

[66] Gurjar M. (2015). Colistin for lung infection: An update. J. Intensive Care.

[67] Aiyer A., Visser S.K., Bye P., Britton W.J., Whiteley G.S., Glasbey T., Kriel F.H., Farrell J., Das T., Manos J. (2021). Effect of N-Acetylcysteine in Combination with Antibiotics on the Biofilms of Three Cystic Fibrosis Pathogens of Emerging Importance. Antibiotics.

[68] Ciacci N., Boncopagni S., Valzano F., Cariani L., Alberti S., Blasi F., Pollini S., Rossolini G.M., Pallecchi L. (2019). In vitro synergism of colistinand N-acetylcysteine against *Stenotrophomonas maltophilia*. Antibiotics.

[69] Conrad C. (2015). Long-term treatment with oral N-acetylcysteine: Affects lung function but not sputum inflammation in cystic fibrosis subjects. A phase II randomized placebo-controlled trial. J. Cyst. Fibros..

[70] Stafanger G., Koch C. (1989). N-acetylcysteine in cystic fibrosis and Pseudomonas aeruginosa infection: Clinical score, spirometry and ciliary motility. Eur. Resp. J..

[71] Dauletbaev N., Fischer P., Aulbach B., Gross J., Kusche W., Thyroff-Friesinger U., Bargon J. (2009). A phase II study on safety and efficacy of high-dose N-acetylcysteine in patients with cystic fibrosis. Eur. J. Med. Res..

[72] Skov M., Pressler T., Lykkesfeldt J., Poulsen E.E., Østrup Jensen P., Johansen H.K., Qvist T., Kræmer D., Høiby N., Ciofu O. (2015). The effect of short-term, high-dose oral N-acetylcysteine treatment on oxidative stress markers in cystic fibrosis patients with chronic *P. aeruginosa* infection—A pilot study. J. Cyst. Fibros..

[73] Dal Negro R., Pozzi E., Cella S.G. (2018). Erdosteine: Drug exhibiting polypharmacy for the treatment of respiratory diseases. Pulm Pharm..

